# Electrolyte Testing Variability Among Critically Ill Children With Viral Bronchiolitis

**DOI:** 10.7759/cureus.105554

**Published:** 2026-03-20

**Authors:** Patrick D Snyder, Kristen Miller, Michael Tchou, Blake Martin

**Affiliations:** 1 Pediatric Critical Care Medicine, Medical University of South Carolina, Charleston, USA; 2 Pediatrics, University of Colorado Anschutz, Aurora, USA; 3 Pediatric Critical Care, University of Colorado Anschutz, Aurora, USA

**Keywords:** high-value care, national trend, pediatric critical care usa, pedictric intensive care unit (picu), viral bronchiolitis

## Abstract

Background

Viral bronchiolitis is the most common indication for pediatric hospitalization for children under 12 months, and admissions for critical bronchiolitis and associated hospital charges continue to rise. Despite this, electrolyte testing practices among children admitted to pediatric intensive care units with bronchiolitis are not well described. This study evaluates the frequency of electrolyte testing and the extent of variation in testing practices across U.S. pediatric ICUs (PICUs).

Methods

We conducted a multicenter, retrospective cohort study of children admitted to PICUs with viral bronchiolitis between January 1, 2017, and December 31, 2021, using the Pediatric Health Information System database. The primary outcomes were (1) the rate of any electrolyte testing and (2) the rate of multiple electrolyte testing during each PICU hospitalization.

Results

Among 23,776 patient encounters, 79% (18,825/23,776) received at least one electrolyte test. Substantial between-hospital variation was observed in the rates of both initial and repeat testing. Hospital-level differences accounted for 32% of the overall variation in any electrolyte testing and 44% of the variation in multiple testing. Among patients who received at least one test (n = 18,825), 80% (15,016) underwent multiple electrolyte tests, while 20% (3,809) received only a single test.

Conclusion

Significant national variation exists in electrolyte testing practices among PICU patients with viral bronchiolitis. These findings highlight substantial heterogeneity in current practice and underscore the need for future studies incorporating clinical context to better define when electrolyte testing is most informative and how testing practices relate to patient characteristics and outcomes.

## Introduction

United States healthcare spending continues to rise at unsustainable rates, totaling $4.7 trillion in 2021 or 18.3% of national GDP [1]. Of this, an estimated $300 billion is spent on children [2]. Approximately one quarter of all healthcare spending is considered waste, with a substantial proportion attributed to overuse or low-value care [3]. Low-value practices, defined as delivery of health care services whose potential benefit does not outweigh their cost or harm [4], remain widespread, yet their true prevalence and impact are poorly understood [5-6]. 

Serum electrolyte testing represents one area of laboratory utilization where testing practices across pediatric populations have increasingly come under evaluation, though these practices have not been well described in children with bronchiolitis requiring pediatric ICU (PICU) admission. When performed more frequently than clinically indicated, electrolyte testing has been associated with measurable harms, including increased healthcare expenditures [7] and iatrogenic anemia [8]. Additional downstream consequences may include increased workload for bedside nurses, reduced patient and family satisfaction related to painful venipuncture [9], and disrupted sleep [10]. Although laboratory testing represents only 3-5% of direct healthcare spending [11], its downstream effects may extend beyond this narrow estimate. Importantly, the prevalence of inappropriate laboratory testing remains poorly defined [7].

Prior work underscores the potential scope of variation in laboratory testing utilization. A multicenter retrospective cohort study by Tchou et al. demonstrated wide variation in electrolyte testing among children hospitalized with common pediatric diagnoses, although that analysis excluded intensive care settings [12]. The existing literature also suggests that pediatric residents receive limited education on value-based care in critical care environments [13] and that overall knowledge of value principles in the PICU remains suboptimal [14]. Notably, no prior studies have evaluated electrolyte testing practices specifically among children admitted to PICUs with viral bronchiolitis.

Despite perceptions that laboratory testing accounts for a small share of PICU hospitalization costs, its cumulative impact may be substantial. Bronchiolitis is the most common cause of hospitalization in infants during the first 12 months of life in developed countries [15]. Over 10 years, Slain et al. observed a 40% increase in bronchiolitis patient volume and a 100% increase in total charges, driven primarily by a 233% increase in PICU admissions and a 160% increase in annual charges among children who did not require invasive mechanical ventilation [16]. These findings highlight the growing burden of critical bronchiolitis and the need to understand drivers of resource use better.

Given the lack of standardized care pathways in critical bronchiolitis [17], this hypothesis-generating study aimed to characterize electrolyte testing practices among children admitted to PICUs with viral bronchiolitis. The primary objective was to describe the frequency of electrolyte testing during PICU hospitalization, including the proportion of encounters with any electrolyte testing and those with multiple electrolyte tests during the admission. A secondary objective was to evaluate variation in testing practices across hospitals and to identify patient- and encounter-level characteristics associated with testing using multilevel modeling approaches. We hypothesized that electrolyte testing practices would vary across institutions and would be associated with patient characteristics, ICU length of stay, and illness severity.

The preliminary results of this study were presented as a poster abstract at the 2023 Pediatric Academic Society Meeting on May 1, 2023.

## Materials and methods

Study design

We conducted a multicenter, retrospective cohort study of children admitted to the PICU with a diagnosis of viral bronchiolitis. The Colorado Multiple Institutional Review Board deemed the study as not human subject research (approval number IRB #23-2343). Data were obtained from the Pediatric Health Information System (PHIS), an administrative database containing inpatient, emergency department, ambulatory surgery, and observation encounter-level data from not-for-profit, tertiary care pediatric hospitals in the United States. These hospitals are affiliated with the Children’s Hospital Association (Lenexa, Kansas, USA). Data quality and reliability are assured through the Children’s Hospital Association and participating hospitals. For external benchmarking, participating hospitals provide discharge/encounter data, including demographics, diagnoses, and procedures. Nearly all participating hospitals submit resource utilization data (e.g., pharmaceuticals, imaging, and laboratory) into PHIS. Data are de-identified upon submission and undergo several reliability and validity checks before inclusion in the database. 

To construct the hospital sample, we identified PHIS hospitals that provided complete billing data for electrolyte testing for each year of the five-year study period. Hospitals with incomplete or missing electrolyte billing data during any study year were excluded. A total of 43 hospitals were included in the sample, with five hospitals excluded based on these criteria. Review of structural characteristics - such as size category, freestanding versus embedded status, and geographic region - indicated that excluded hospitals were generally comparable to included hospitals, reducing the likelihood of major structural selection bias (Appendix 1). However, differences in hospital billing or charge-capture practices within administrative datasets like PHIS could influence the recorded frequency of laboratory testing.

Study population

We included all encounters for patients younger than 2 years admitted to a PICU between January 1, 2017, and December 31, 2021, with a discharge diagnosis of viral bronchiolitis. Encounters involving invasive mechanical ventilation were excluded to create a more clinically homogeneous cohort, as ventilated patients often undergo routine laboratory monitoring related to ventilator management, sedation, and blood gas assessment. Including these encounters could substantially alter testing patterns and obscure variation in electrolyte testing practices among non-ventilated PICU bronchiolitis patients.

Measures

The primary outcomes of interest were the rate of any electrolyte testing, defined as the proportion of PICU hospitalizations during which at least one electrolyte test was performed, and the rate of multiple electrolyte testing, defined as the proportion of PICU hospitalizations with more than one electrolyte test performed. 

Because PHIS reports laboratory charges at the calendar-day level, the multiple testing outcome only captured tests performed on separate days; repeat tests performed within the same calendar day could not be differentiated. Point-of-care electrolyte tests were not included. ICU length of stay (LOS) was examined as a secondary outcome in relation to electrolyte testing frequency.

Exposure variables included complex chronic condition flag (CCC) [18], use of electrolyte-altering medications (diuretics, desmopressin, inhaled beta agonists), ICU LOS, severity of illness, discharge year (2017-2021), sex, race (Non-Hispanic White, Asian, Hispanic, Multiracial, Non-Hispanic Black, or Other), and payor category (Commercial, Medicaid, Other Government, Self-Pay, or Other Payor). Severity of illness was classified using the 3M™ All Patient Refined Diagnosis-Related Groups (APR-DRG; Health Information Systems, Salt Lake City, USA) [19] severity measure, an administrative risk adjustment tool commonly used in PHIS datasets, though it may not fully capture acute physiologic severity among critically ill ICU patients.

Statistical methods

Hospital-level summary statistics were produced using frequencies and proportions (n, %), or medians with interquartile range (IQR) and ranges, as appropriate. We performed discharge-level summary statistics, stratified by whether an electrolyte test occurred during the hospitalization. 

We used generalized mixed-effects logistic regression models with a logit link to identify independent predictors of (1) any electrolyte testing and (2) multiple electrolyte testing (defined as electrolyte testing on ≥2 hospital days during the same PICU hospitalization). A random intercept for hospital was included to account for clustering and to estimate the degree of between-hospital variation. No patient encounters were excluded from the regression analyses due to missing predictor variables. For categorical variables with more than two levels, p-values from Type III Wald Chi-Square tests were reported. The intraclass correlation coefficient (ICC) quantified the proportion of variance attributable to between-hospital differences.

Hospital-specific random effects and 95% confidence intervals were estimated using the Gelman simulation technique (500 simulations) and plotted to visualize variation in testing across hospitals. The multiple-testing model included only encounters in which at least one electrolyte test was performed. Differences in ICU LOS between testing groups (any vs. multiple tests) were assessed using the Wilcoxon rank-sum test.

Analyses were conducted using R version 4.2.2 (R Foundation for Statistical Computing, Vienna, Austria) with the lme4 and merTools packages. Model performance was assessed through ICC, fixed-effect precision, and inspection of hospital-level random effects. Model diagnostics included evaluation of model convergence, assessment of multicollinearity among predictors, and inspection of the distribution of random effects. Effect sizes and 95% confidence intervals are reported for all predictors. No major violations of model assumptions were identified.

## Results

We excluded five hospitals due to incomplete electrolyte billing data. The final cohort included 23,776 patient encounters from 43 hospitals. Overall, 79.2% (18,825/23,776) of encounters received at least one electrolyte test during the PICU hospitalization.

Hospital-level use of electrolyte testing

**Figure 1:**
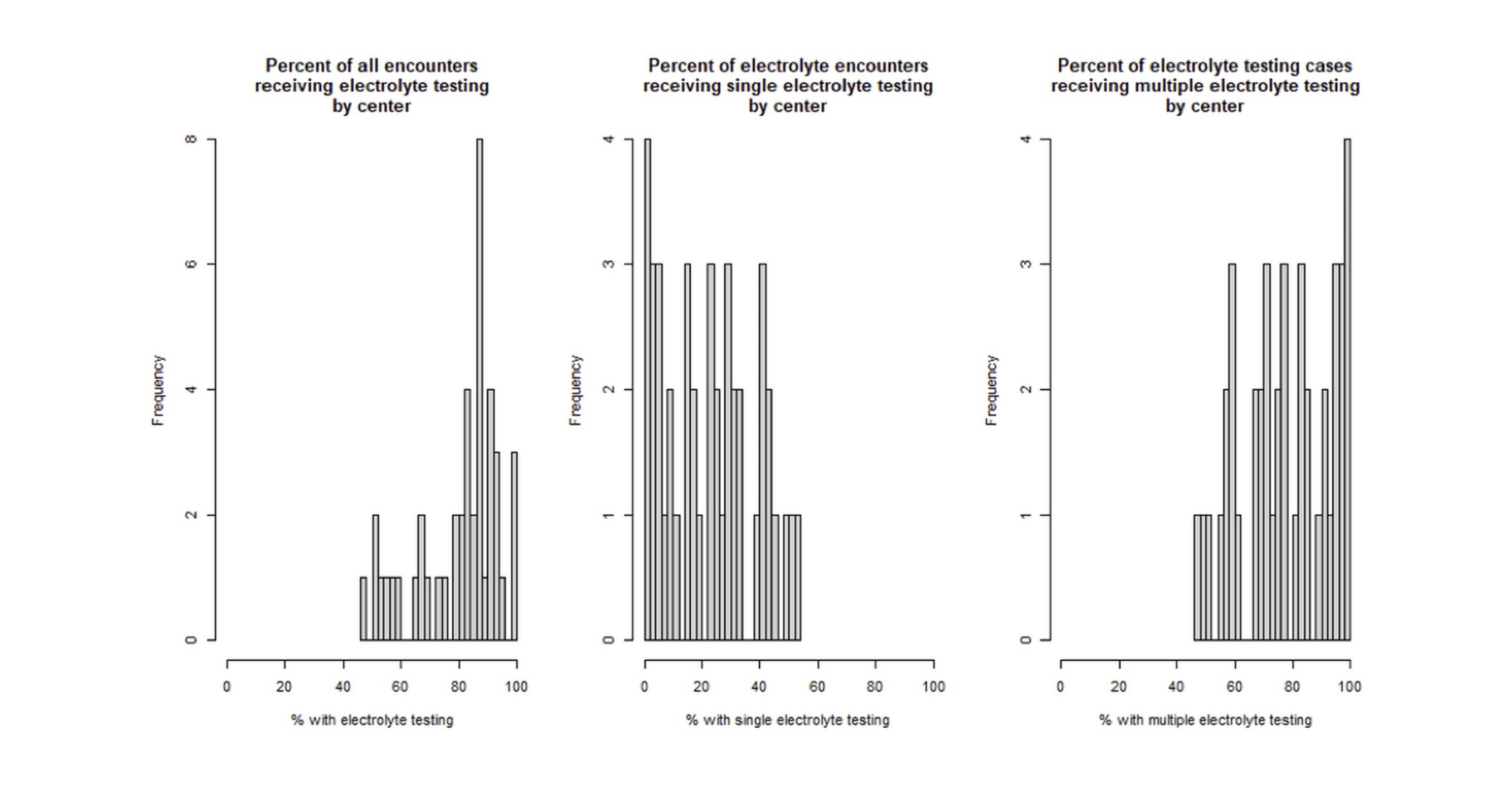
Hospital Testing Rates Histograms reported for rates of testing by center, including percent of encounters receiving any electrolyte test, a single electrolyte test, and multiple electrolyte tests.

There was substantial between-hospital variation in both initial and multiple electrolyte testing (Figure 1). Across hospitals, the proportion of PICU hospitalizations with any electrolyte testing ranged from 47% to 100%. Among patients who underwent electrolyte testing, the proportion receiving only a single test ranged from 0% to 54% across hospitals, while the proportion receiving multiple tests ranged from 46% to 100%. Of all patients who received at least one electrolyte test (n = 18,825), 80% (15,016) received more than one test. The highest values (e.g., 100%) potentially reflect hospitals with relatively small numbers of eligible encounters. Hospital-level estimates should therefore be interpreted in the context of varying institutional bronchiolitis case volumes. The mixed-effects modeling framework accounts for clustering by hospital when estimating predictors of testing.

Independent predictors of any electrolyte testing

**Figure 2:**
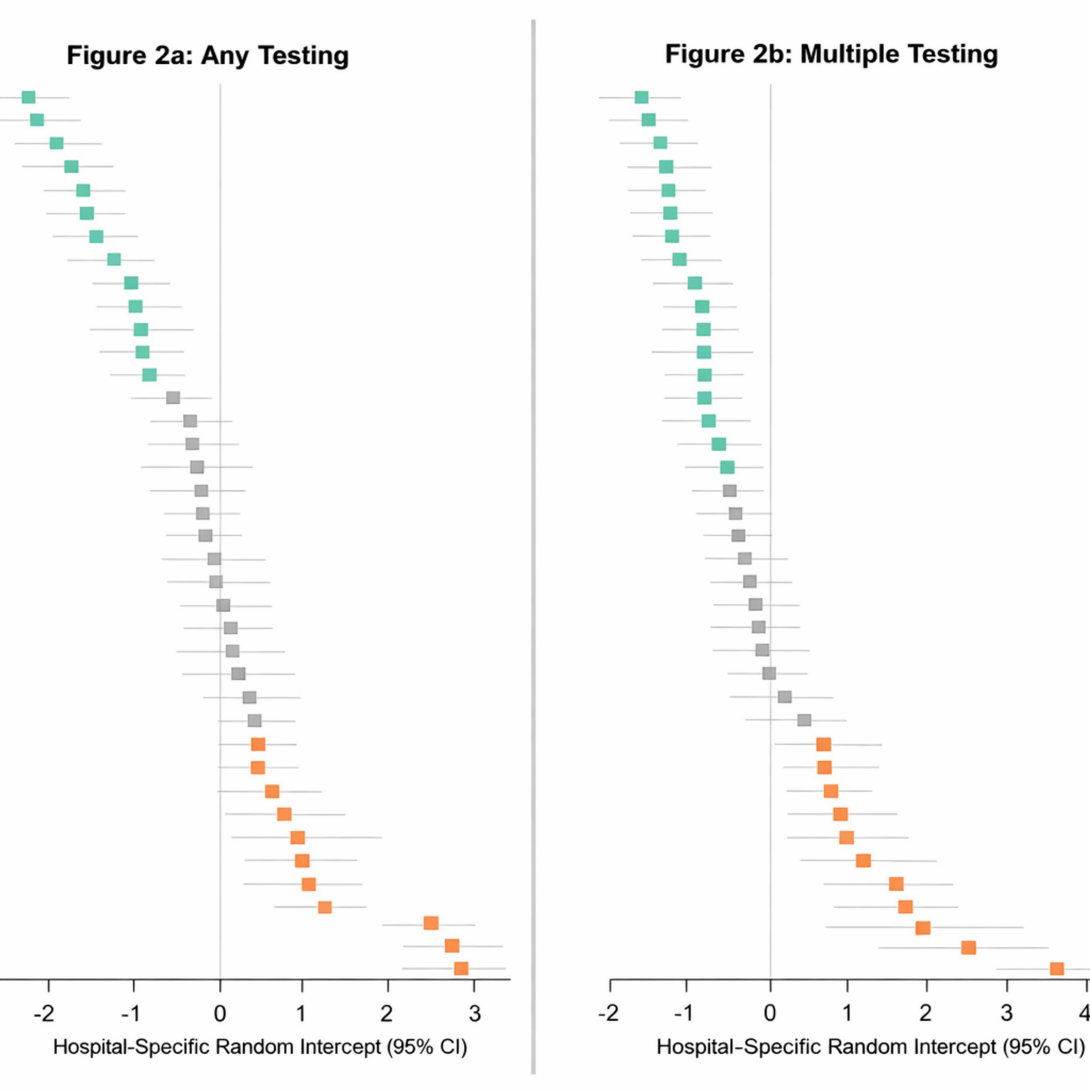
Hospital-Level Proportion of Variability (a) Any testing: After adjusting for patient-level factors and characteristics in Table 2, there were 14/43 (33%) hospitals with significantly lower than average electrolyte testing rates (green dots) and 12/43 (30%) hospitals with significantly higher than average testing rates (orange dots). The vertical line represents the average rate of testing. Hospitals in grey are not significantly different than the average. There were three hospitals with rates considerably higher than the average. The adjusted intraclass correlation coefficient (ICC) of this model is 0.32, indicating that between-hospital differences account for 32% of the differences in electrolyte testing rates. (b) Multiple testing: After adjusting for patient-level factors listed in Table 2, there were 19/43 (44%) hospitals with significantly lower than average multiple electrolyte testing rates (green dots) and 12/43 (28%) hospitals with significantly higher than average multiple testing rates (orange dots). The vertical line represents the average rate of multiple testing, and hospitals in grey are not significantly different than the average. The adjusted intraclass correlation coefficient (ICC) of this model is 0.44, indicating that between-hospital differences account for 44% of the differences in electrolyte testing rates.

After adjusting for patient-level characteristics (Table 1), meaningful variability persisted across hospitals. For any electrolyte testing (Figure 2a), 14 of 43 hospitals (33%) had significantly lower-than-average adjusted testing rates, while 12 of 43 hospitals (30%) had significantly higher-than-average rates. The adjusted intraclass correlation coefficient (ICC) was 0.32, indicating that 32% of the variation in electrolyte testing was attributable to between-hospital differences. Intraclass correlation coefficients (ICC) were used to quantify the proportion of unexplained variability in electrolyte testing attributable to clustering at the hospital level rather than patient-level differences. Higher ICC values indicate greater between-hospital variation in testing practices after accounting for measured patient characteristics. These estimates reflect variability associated with hospital-level clustering and should not be interpreted as evidence of causal hospital-level effects.

**Table 1:** Cohort-Wide Summary Statistics The rates of electrolyte testing, as well as single- and multiple-electrolyte testing, were calculated for each variable and displayed using frequencies and proportions (n, %). Days in ICU are reported as median (interquartile range).

Characteristic	No Testing (n=4912)	Any Testing (n=18825)	Multiple Tests (n=15016)	Single Test (n=3809)
Discharge year				
2017	1322 (27%)	4149 (22%)	3309 (22%)	840 (22%)
2018	757 (15%)	4610 (24%)	3629 (24%)	981 (26%)
2019	1004 (20%)	4601 (24%)	3742 (25%)	859 (23%)
2020	365 (7%)	2014 (11%)	1576 (10%)	438 (11%)
2021	1464 (30%)	3451 (18%)	2760 (18%)	691 (18%)
Gender				
Female	1973 (40%)	7761 (41%)	6248 (42%)	1513 (40%)
Male	2937 (60%)	11054 (59%)	8764 (58%)	2290 (60%)
Unknown	2 (0%)	10 (0%)	4 (0%)	6 (0%)
Race category				
Asian	132 (3%)	536 (3%)	443 (3%)	93 (2%)
Hispanic	946 (19%)	3622 (19%)	2804 (19%)	818 (21%)
Multiracial	49 (1%)	232 (1%)	183 (1%)	49 (1%)
Non-Hispanic Black	1186 (24%)	4301 (23%)	3425 (23%)	876 (23%)
Non-Hispanic White	2055 (42%)	8441 (45%)	6813 (45%)	1628 (43%)
Other	319 (6%)	1101 (6%)	878 (6%)	223 (6%)
Unknown	225 (5%)	592 (3%)	470 (3%)	122 (3%)
Payor category				
Commercial	1683 (34%)	5784 (31%)	4489 (30%)	1295 (34%)
Government: Medicaid	2942 (60%)	11770 (63%)	9493 (63%)	2277 (60%)
Government: Other	94 (2%)	332 (2%)	250 (2%)	82 (2%)
Other Payor	109 (2%)	339 (2%)	298 (2%)	41 (1%)
Self-Pay	65 (1%)	341 (2%)	254 (2%)	87 (2%)
Unknown	19 (0%)	259 (1%)	232 (2%)	27 (1%)
Days in ICU	2 (1, 2)	2 (2, 4)	3 (2, 5)	2 (1, 3)
Severity level				
1	693 (14%)	1392 (7%)	1003 (7%)	389 (10%)
2	642 (13%)	2359 (13%)	1815 (12%)	544 (14%)
3	3057 (62%)	10073 (54%)	7804 (52%)	2269 (60%)
4	520 (11%)	5001 (27%)	4394 (29%)	607 (16%)
Complex chronic condition				
No	3976 (81%)	13197 (70%)	10165 (68%)	3032 (80%)
Yes	936 (19%)	5628 (30%)	4851 (32%)	777 (20%)
Drug Exclusion				
No	2162 (44%)	13048 (69%)	10562 (70%)	2486 (65%)
Yes	2750 (56%)	5777 (31%)	4454 (30%)	1323 (35%)

Several discharge-level characteristics were independently associated with the odds of receiving any electrolyte test (Table 2). Higher likelihood of electrolyte testing was observed among patients with longer ICU length of stay, greater severity of illness (compared with severity level 1), discharge years 2018-2020 (vs. 2017), and Medicaid or other payer types compared with commercial insurance. In contrast, patients without exposure to electrolyte-altering medications (i.e., those not receiving diuretics, desmopressin, or inhaled beta agonists) had lower odds of electrolyte testing.

**Table 2:** Models Adjusting for Patient-Level Characteristics Using discharge-level data, a generalized mixed model using the fixed effect parameters was used to assess for independent predictors of any electrolyte testing, as well as for multiple electrolyte tests. The model only included patients with at least one electrolyte test.

Clinical or Demographic Parameter	Any testing		Multiple testing	
	OR (95% CI)	P value	OR (95% CI)	P value
Complex Chronic Condition Flag	1.06 (0.96, 1.18)	0.223	1.43 (1.30, 1.60)	<0.001
Drug Exclusion	0.37 (0.34, 0.40)	<0.001	0.84 (0.78, 0.93)	<0.001
Total Days in ICU (log scale)	2.29 (2.15, 2.45)	<0.001	2.16 (2.02, 2.30)	<0.001
Severity Level (ref=1)				
2	1.58 (1.35, 1.85)	<0.001	1.10 (0.94, 1.32)	<0.001
3	1.77 (1.54, 2.03)		1.33 (1.16, 1.57)	
4	3.28 (2.75, 3.90)		2.06 (1.73, 2.51)	
Discharge Year (ref=2017)				
2018	2.10 (1.87, 2.37)	<0.001	0.82 (0.73, 0.93)	<0.001
2019	1.42 (1.27, 1.59)		0.83 (0.74, 0.95)	
2020	1.81 (1.54, 2.11)		0.67 (0.57, 0.79)	
2021	0.90 (0.81, 1.01)		1.10 (0.98, 1.26)	
Payor (ref=commercial)				
Government: Medicaid	1.12 (1.02, 1.23)	0.0129	1.04 (0.94, 1.15)	0.1286
Government: Other	0.88 (0.66, 1.17)		0.89 (0.67, 1.20)	
Other Payor	1.51 (1.11, 2.05)		1.62 (1.19, 2.45)	
Self-Pay	1.23 (0.90, 1.68)		0.88 (0.64, 1.18)	
Race (ref=Non-Hispanic White)				
Asian	1.15 (0.91, 1.47)	0.0896	1.28 (1.00, 1.67)	0.6382
Hispanic	1.03 (0.92, 1.16)		1.00 (0.89, 1.14)	
Multiracial	1.15 (0.80, 1.64)		1.03 (0.72, 1.48)	
Non-Hispanic Black	0.88 (0.79, 0.98)		1.00 (0.89, 1.12)	
Other	0.98 (0.83, 1.16)		1.04 (0.88, 1.26)	
Male (ref=Female)	0.93 (0.86, 1.00)	0.0555	0.89 (0.82, 0.97)	0.0069

Independent predictors of multiple electrolyte testing

Among patients who received at least one test, the proportion who underwent multiple electrolyte tests also varied substantially across hospitals. After adjustment for patient-level factors, 19 of 43 hospitals (44%) had significantly lower-than-average rates of multiple testing, while 12 of 44 hospitals (27%) had significantly higher-than-average rates (Figure 2b). The adjusted ICC was 0.44, indicating that 44% of the variation in multiple electrolyte testing rates was attributable to between-hospital differences.

Several discharge-level characteristics were also associated with the likelihood of receiving multiple electrolyte tests (Table 2). Greater illness severity was independently associated with higher odds of multiple testing, including severity level 3 (OR 1.33, 95% CI 1.16-1.57) and severity level 4 (OR 2.06, 95% CI 1.73-2.51) compared with severity level 1. Longer ICU length of stay was also associated with increased odds of repeat testing. In contrast, patients without exposure to electrolyte-altering medications had lower odds of receiving multiple electrolyte tests. Additional associations, including discharge year and payer category, are shown in Table 2.

ICU length of stay

There was a strong, significant association between ICU LOS and electrolyte testing. Longer ICU LOS was associated with higher odds of both any electrolyte testing (OR 2.29, 95% CI 2.15-2.45, p<0.001) and multiple testing (2.16, 2.02-2.30, p<0.001) (Table 2).

## Discussion

In this large, multicenter study, we identified significant variations in both initial and multiple electrolyte testing rates across PICUs, even after accounting for patient-level factors. Between-hospital differences accounted for 32% of the variability in any electrolyte testing and 44% of the variability for multiple electrolyte tests. While this degree of variation suggests heterogeneity in care delivery, conclusions regarding overuse must be interpreted cautiously. Because we lacked access to clinical indications, laboratory values, or downstream management decisions, our findings should be viewed as descriptive and hypothesis-generating rather than determinative of low-value care.

The retrospective nature of the study further limits causal inference; however, the persistence of variation after restricting analyses to a single diagnosis and adjusting for key clinical factors raises the possibility that at least some observed differences may represent unintended variation rather than clinically justified practice. Intended variation [20] - such as electrolyte monitoring in response to electrolyte-altering therapies, evolving clinical status, or known abnormalities and risk factors - may represent appropriate, value-based care. In contrast, unintended variation may arise from local practice norms, institutional culture, or default ordering behaviors and may have the potential to contribute to inefficiencies and excess cost. Our data do not allow these scenarios to be distinguished. Nonetheless, even if a portion of the observed variability reflects discretionary testing without clear clinical benefit, targeted efforts to standardize practice could improve resource utilization among children hospitalized with critical bronchiolitis.

Several predictors of electrolyte testing aligned with clinical expectations. Longer ICU length of stay, higher illness severity, and the presence of a complex chronic condition were associated with increased odds of testing, likely reflecting clinicians’ heightened vigilance in higher-risk patients. In contrast, some temporal findings were less intuitive. Compared with 2017, discharge years 2018-2020 were associated with higher odds of initial electrolyte testing but lower odds of multiple testing. These patterns may reflect evolving laboratory stewardship initiatives, increased awareness of test overutilization, or broader institutional shifts toward value-based care. The COVID-19 pandemic may also have contributed to these trends. Public health mitigation measures substantially altered the epidemiology of bronchiolitis and other respiratory viral infections, including reduced respiratory syncytial virus (RSV) circulation during 2020, followed by atypical seasonal resurgence and shifts in the age distribution and severity of hospitalized children [21]. In one retrospective time series study across a large health system serving women and children, some laboratory tests showed an 80% decline in utilization during the mandatory pandemic lockdown [22]. Changes in case mix, along with pandemic-related disruptions to ICU workflow and staffing, may have influenced testing practices in ways not captured by administrative data. While these explanations remain speculative, they highlight the complexity of interpreting temporal trends in retrospective datasets.

Efforts to reduce low-value care increasingly emphasize thoughtful evaluation of routine laboratory testing. In the setting of critical bronchiolitis, particularly among lower-risk children admitted to the PICU, variability in laboratory utilization, which includes electrolyte testing, may represent a potentially modifiable contributor to healthcare costs. Slain et al. documented marked increases in both PICU admissions and associated hospital charges over a decade, highlighting the importance of addressing potentially modifiable drivers of resource use [16]. Existing studies evaluating electrolyte testing across pediatric populations demonstrate that such tests infrequently lead to changes in management [23-25]. However, these findings largely derive from non-critical care populations, and it remains unclear whether they apply to children with severe bronchiolitis requiring PICU admission. Within this context, our findings may help inform future quality improvement initiatives aimed at aligning electrolyte testing with clinical risk, while preserving appropriate monitoring for patients with clear indications. Notably, improvement collaboratives have demonstrated the potential for meaningful reductions in laboratory utilization without adverse effects. For example, a multisite effort by Coe et al. achieved a 13% reduction in electrolyte testing on hospital medicine services [26], while a single-center initiative by Tchou et al. demonstrated a 35% reduction in electrolyte testing and a 29% reduction in hospital charges without increasing adverse events [27].

Importantly, our results should not be interpreted as suggesting that electrolyte testing is unnecessary in critically ill children with bronchiolitis. Rather, they highlight the need to identify subgroups in whom monitoring is most likely to provide clinical value. Prior studies demonstrate that electrolyte abnormalities occur with meaningful frequency in moderate to severe bronchiolitis and may be associated with adverse outcomes in selected populations. Hyponatremia, typically mild, has been shown to occur commonly in younger infants, particularly those under six months of age [28], and has been associated with hypotonic fluid administration and longer hospital length of stay [29]. In addition, hypophosphatemia is prevalent in severe bronchiolitis and has been associated with prolonged duration of mechanical ventilation [30]. Together, these findings suggest that risk-stratified electrolyte testing, informed by factors such as age, illness severity, respiratory support, and fluid management, may be clinically useful for specific higher-risk subgroups. In contrast, routine electrolyte testing among children with less severe bronchiolitis or without established risk factors may offer limited benefit and warrants further evaluation. Future studies incorporating granular clinical data will be essential to distinguish appropriate clinical responsiveness from potentially modifiable practice variation and to inform the development of standardized, risk-based testing strategies.

This study has several important limitations. First, the cohort consisted exclusively of U.S.-based tertiary and quaternary children’s hospitals participating in PHIS, which may limit generalizability to community hospitals, mixed adult-pediatric ICUs, or international settings. Second, the use of administrative data limited access to laboratory results and important clinical context typically available in the electronic medical record, including laboratory test timing, intravenous fluid composition, electrolyte replacement, feeding status, urine output, and medication dosing or frequency, all of which may influence decisions to obtain electrolyte testing. These omissions likely contributed to residual confounding. Additionally, PHIS captures charges at the calendar-day level, preventing identification of multiple tests performed within the same day and likely underestimating the true frequency of repeated electrolyte testing. Furthermore, illness severity was measured using APR-DRG classification, which is derived from administrative coding and may not fully reflect acute physiologic severity in critically ill patients. Finally, because ICU length of stay was included as a predictor of testing, the temporal relationship between testing and hospitalization duration cannot be fully disentangled. Patients with longer ICU stays have more opportunities for laboratory testing, raising the possibility of reverse causality.

Encounters involving invasive mechanical ventilation were excluded to focus on a more clinically homogeneous population of PICU patients with bronchiolitis. Ventilated patients often undergo frequent laboratory monitoring as part of routine ventilator management and sedation practices, which may substantially influence testing patterns. However, this exclusion may introduce selection bias and limit the generalizability of our findings to the most critically ill bronchiolitis patients. Future studies should evaluate electrolyte testing practices specifically within this population.

Finally, while our models adjusted for several important covariates, unmeasured clinical factors - such as fluid balance, renal function, feeding strategy, or response to prior electrolyte results - may still influence testing decisions. Sensitivity analysis stratified by illness severity or CCC status would further strengthen interpretation and represent an important opportunity for future research.

## Conclusions

In this large, multicenter study of PICU patients with viral bronchiolitis, we identified substantial and unexplained variation in electrolyte testing practices across hospitals, even after adjustment for patient characteristics and illness severity. While some variation is expected based on clinical acuity, the magnitude of between-hospital differences highlights heterogeneity in current practice patterns. Future work incorporating detailed clinical data, including electrolyte results and treatment context, as well as studies focused on the most severely ill children, will be essential to determine when electrolyte testing is most appropriate and whether risk-stratified or standardized approaches can safely support improved resource use while maintaining patient safety.

## Appendices

Appendix 1: hospital-level characteristics of the analytic sample

**Table 3:** Hospital-Level Characteristics of the Analytic Sample This table reports the structural characteristics of the 43 hospitals included in the final cohort, as well as the five excluded hospitals. Characteristics include bed-size category (small, medium, large), hospital type (“F” freestanding pediatric-only facility vs. “E” embedded within a general or academic medical center), Part of a System, and geographic region. These variables were selected to allow comparison of institutional features between included and excluded hospitals and to help interpret the extent to which observed variation in electrolyte testing may reflect underlying structural or organizational differences.

Hospital	Staffed Beds	Hospital Type	Part of System	Region
1	Large	F	No	Midwest
2	Medium	F	Yes	Midwest
3	Medium	E	No	South
4	Large	F	No	South
5	Large	F	No	Northeast
6	Medium	E	Yes	South
7	Large	E	Yes	South
8	Medium	E	Yes	Midwest
9	Medium	E	Yes	Midwest
10	Large	F	No	Midwest
11	Large	F	No	Midwest
12	Medium	E	Yes	Midwest
13	Large	F	No	Midwest
14	Large	E	Yes	South
15	Large	F	No	West
16	Large	E	No	South
17	Medium	F	No	Northeast
18	Large	F	No	South
19	Medium	E	Yes	South
20	Large	F	Yes	Midwest
21	Large	F	Yes	Midwest
22	Medium	F	Yes	West
23	Large	F	Yes	South
24	Medium	F	No	West
25	Large	F	Yes	South
26	Large	F	Yes	South
27	Medium	F	Yes	Midwest
28	Large	E	Yes	Midwest
29	Large	F	No	South
30	Medium	E	Yes	Northeast
31	Medium	F	No	South
32	Medium	E	Yes	West
33	Medium	F	No	Midwest
34	Large	F	Yes	West
35	Large	E	Yes	West
36	Large	F	Yes	Northeast
37	Large	F	Yes	Northeast
38	Large	F	Yes	West
39	Large	F	Yes	West
40	Large	F	No	West
41	Large	E	Yes	Midwest
42	Large	F	Yes	South
43	Large	F	No	South
Excluded Hospitals				
44	Large	F	Yes	South
45	Medium	E	Yes	Northeast
46	Large	F	Yes	South
47	Large	F	No	West
48	Medium	E	Yes	Northeast

 Appendix 2: Pediatric Health Information System (PHIS) hospital charge codes used to define the study population

**Table 4:** Pediatric Health Information System (PHIS) Hospital Charge Codes Used to Define the Study Population

Query Filter	Code	Title
Lab Test Code	313110	Random glucose
	311290	Potassium
	311291	Sodium
	311211	Ionized calcium
	310108	8-test chemistry profile
	311250	Magnesium
	311280	Phosphorus
	311220	Chloride
	310114	14-test chemistry profile
	318135	Creatinine
	310300	Kidney profile
	311210	Calcium
	311297	Electrolyte profile
	310109	8-test chemistry profile - ionized calcium
Principal Diagnosis (ICD)	4661	Acute bronchiolitis
	46611	Acute bronchiolitis due to respiratory syncytial virus (rsv)
	46619	Acute bronchiolitis due to other infectious organisms
	J210	Acute bronchiolitis due to respiratory syncytial virus
	J211	Acute bronchiolitis due to human metapneumovirus
	J218	Acute bronchiolitis due to other specified organisms
	J219	Acute bronchiolitis, unspecified
Generic Drug Code	191100	Diuretics unspecified
	191190	Diuretic combinations
	191199	Other specified diuretics
	191135	Furosemide
	151475	Desmopressin acetate
	181315	Albuterol sulfate and ipratropium bromide
	181231	Levalbuterol HCl
	181211	Albuterol (sulfate)
